# A state observer-based robust control method for perturbation-containing nonlinear discrete interconnected Systems

**DOI:** 10.1371/journal.pone.0320912

**Published:** 2025-04-29

**Authors:** Yanxiu Sun, Yu Wen, Hong Li, Ying Du

**Affiliations:** 1 Basic Course Department, Shenyang Institute of Technology, Fushun, China; 2 College of Sciences and Mathematics, Handan University, Handan, China; University of Shanghai for Science and Technology, CHINA

## Abstract

In this study, the problem of the robust control of interconnected systems was investigated. A controller design method based on a state observer is proposed for nonlinear discrete interconnected systems with disturbance terms. In the proposed method, the observer and controller of the interconnected systems are designed separately, and sufficient conditions for the existence of the gain matrix are provided. In the design of the state observer, considering the convergence rate of the observation error, the performance of the state observer was improved by adding constraints. The influence of the external disturbance of the system on the state estimation is reduced by introducing a performance index, which provides the necessary theoretical basis for the design of the controller. In the controller design, based on the disk stability lemma and control theory, sufficient conditions for the existence of the controller are provided, and robust stabilization of the feedback closed-loop system was realized. In this study, the separate design method of the controller was compared with the centralized design method, which demonstrated the superiority of the separate design of the observer and controller. Finally, a numerical example is discussed, in which the separation design method of the robust controller was tested, and the effectiveness of the method was verified.

## 1. Introduction

State feedback control is an important research topic in modern control theory. In general, state feedback control is performed on the premise that the state information of the control system is completely available; however, for technical or economic reasons, the system state is often difficult or impossible to measure. A system state observer can achieve robust estimation of state variables on the basis of control input and output, and solve the problem that the state cannot be measured directly through reconstruction. State observer plays an important role in fault tolerant control, fault estimation and diagnosis, finite time predictive control, multi-agent cooperative control, etc. It provides a necessary theoretical basis for realizing effective and fault diagnosis of control system.

In recent years, the theory of discrete systems has been widely used in biological systems, communication systems, computer systems, data-processing systems, and other industrial and social fields. In the study of discrete systems, problems such as the observer design method [1–12], fault estimation and diagnosis based on a state observer [13–16], and observer-based feedback control [17–21] have attracted significant attention and achieved certain theoretical achievements.

Discrete systems have made certain achievements in observer-based fault diagnosis. Dai et al. [13] designed a fault observer for discrete systems with faults to realize robust fault estimation of the system, and fault-tolerant control of the discrete system was realized on the basis of the fault observer. Zhu et al. [14] studied discrete systems with actuator faults and external disturbances, and the purpose of interval estimation and diagnosis of actuator faults was realized by constructing a dimension-reduction observer. Li et al. [15] proposed a design method for a centrosymmetric polycellular set member fault detection observer for a class of discrete systems to effectively determine whether a fault occurs. Sun and Li [16] studied sensor fault estimation for a class of nonlinear interconnected systems, and proposed an observer-based fault estimation method for interconnected systems.

Discrete systems yield more useful results for observer-based robust control. Lu and Yu [17] applied the differential mean value theorem and linear parametric system theory to develop a design method for an observer-based robust controller. Nguyen et al. [18] proposed a less conservative and robust control method based on a state observer for a class of discrete systems with unilateral Lipschitz conditional nonlinear terms. This method was extended by Nguyen et al. [19], and a robust control method for nonlinear discrete systems with uncertain terms was further proposed. Sun and Long [20] presented a robust control method for nonlinear discrete systems that considers the convenience of the gain matrix solution, accuracy of state estimation, and rapidity of feedback control, and extended the method to the singular system form. Xu et al. [21] studied a class of discrete-time multi-agent systems, designed a disturbance observer based on the Lyapunov stability theory, and realized the purpose of state feedback and output feedback control.

An interconnection system is a complex, large-scale system composed of several subsystems connected by interconnection items. The scale and complexity of systems involved in production and life are increasing. Interconnected systems have been widely used in applications such as power systems, urban transportation systems, and unmanned aerial systems, and have become a key research topic [22–26]. Mu et al. [24] studied a robust control problem for a class of continuous nonlinear interconnected systems, and proposed an observer-based control method. Xie et al. [25] proposed a type of predictive control problem for discrete-time interconnected systems, and provided the guaranteed conditions for the existence of predictive controllers in interconnected systems. Farbood et al. [26] proposed a new method of system predictive control, which takes new ellipsoidal constraints into account in the controller design process, and improves the feasibility of optimization problems.

At present, the problems of nonlinear interconnected system observer design and fault estimation and diagnosis or robust control based on interconnected system observer are increasing, and relevant theoretical research results are gradually applied to many fields such as unmanned aerial system and power system. Discrete interconnection system is widely used in digital signal processing, digital control system and so on. Robust control methods based on state observers have rarely been reported for nonlinear discrete interconnected systems. In this study, the designs of a system observer and observer-based feedback control were investigated for nonlinear discrete interconnected systems with disturbances, and a robust control method for interconnected systems based on a state observer is proposed. Considering the influence of external disturbances and the convergence time of estimation errors, sufficient conditions for the existence of the gain matrix are provided in the form of linear matrix inequalities, and the controller design is presented. Finally, the superiority and effectiveness of the control method were tested using numerical examples.

Multi-agent systems are widely used in complex task coordination and distributed control, which has attracted great attention from scholars. Consistency control is one of the core problems in the research of multi-agent system. The purpose is to design a suitable strategy to make all agents in the system reach the desired state.

In this paper, a robust control method based on observer is presented to achieve robust stabilization of the interconnected system. This method can be used as reference for the observe-based multi-agent system consistency control method.

The main contributions of this study are as follows.

1. A state-observer design scheme is proposed for a class of discrete nonlinear interconnected systems. By introducing one performance index and stability margin, the performance of the interconnected system state observer was improved, providing the basis for robust control of the system. Considering the actual environment of a system, studying an interconnected system with nonlinear terms and external interference renders the designed observer more robust and practical.
2. Considering the complexity of the interconnected system and avoiding the influence of the interconnected terms on the design of the observer and controller, the local dynamic system with the estimation error and feedback control of each subsystem is described in a global form, which is more convenient for the design and calculation of the Lyapunov function and can be based on the design methods of normal system observers and controllers. Sufficient conditions for the existence of each subsystem observer and controller in the interconnected system are provided.
3. In the process of controller design of nonlinear discrete interconnected systems, the Lyapunov stability theory and disk stability lemma are used to constrain the pole placement of the system, ensuring the stability of the estimation error system and closed-loop control system. The observer and controller design are designed separately in the proposed method, so that the observer and controller designs have greater freedom, which is conducive to realizing the robust stabilization of the feedback system.

The rest of this article is organized as follows. In the “Problem description” section, the structure of a class of nonlinear discrete interconnected systems with perturbations is described and the assumptions required for interconnected systems are presented. In “State observer design for interconnected systems,” an observer design method for discrete interconnected systems is presented, which provides a basis for the feedback control of interconnected systems. In “Controller design of an interconnected system based on observed state,” a robust control method for discrete interconnected systems based on a state observer is proposed. The observer and controller are designed separately, and sufficient conditions for the existence of the designed observer and controller are provided. In “Simulation examples and analysis,” the experiments performed to test the proposed robust control method for interconnected systems are discussed using numerical examples, verifying the effectiveness and feasibility of the proposed method. The final section summarizes the advantages of the proposed method and provides directions for future research.

**Notation:** I represents an identity matrix of appropriate dimensions, and 0 represents scalar zero or a zero matrix of appropriate dimensions; ‖·‖ denotes the standard Euclidean norm or its induced norm; for any matrix A,A>0denotes that it is positive definite; ${A^{+},\;A}^{T}$and $A^{-1}$are generalized inverse and the transpose and inverse of matrix A, respectively; and * represents the symmetric term of the symmetric matrix.

## 2. Problem description

Consider the following nonlinear discrete interconnected system:


$$\left\{ \begin{array}{c} @lx_{i}(k+1)=A_{i}x_{i}(k)+B_{i}u_{i}(k)+g\left[x_{i}(k)\right]+\sum_{\begin{array}{c} j=1\\ j\neq i \end{array}}^{N}H_{ij}x_{j}(k)+D_{i}d_{i}(k)\;\\ y_{i}(k)=C_{i}x_{i}(k),i=1,2,\cdots ,N.\; \end{array}\;\right.\;$$ (1)

where $x_{i}(k)\in R^{n},{u_{i}(k)\in R^{m},y}_{i}(k)\in R^{p}$ and $d_{i}(k)\in R^{l}$ are the state, control inputs, outputs, and system disturbances of the i-*th* subsystem, $g\left[x_{i}(k)\right]$is the system nonlinear term, and $A_{i}{,B}_{i},\;C_{i},D_{i}$are the coefficient matrices with appropriate dimensions in the i-*th* subsystem, $H_{ij}$ is the interconnection matrix between the i-*th* sub-system and the j-*th* sub-system in the interconnected system.

***Remark 1:*** Interconnected system is a large system composed of several subsystems connected by coupling items. These subsystems are interrelated in different ways through the interconnection items in the system to achieve the overall function of the system. The relationship between the subsystems is mainly reflected in the connection and communication between them, this paper mainly studies the robust control method of nonlinear discrete-time interconnected large system.

***Assumption 1*** The subsystems of the interconnected system (1) are observable in matrix $\left[A_{i}\;C_{i}\right]$ and controllable in matrix $\left[A_{i}\;B_{i}\right]$.

***Assumption 2*** The nonlinear term $g\left[x_{i}(k)\right]$ in each subsystem of the interconnected system (1) satisfies the Lipschitz condition:


$$\left\|g\left[x_{i}(k)\right]-g\left[{\hat{x}}_{i}(k)\right]\right\|\leq L_{g_{i}}\left\|x_{i}(k)-{\hat{x}}_{i}(k)\right\|,i=1,2,\cdots ,N.$$ (2)

Where $L_{g_{i}}$ is the Lipschitz constant, $\forall x_{i}(k),{\tilde{x}}_{i}(k)\in R^{n}$.

***Lemma 1*** (Schur complement lemma) [2]: The following conditions are equivalent for symmetric matrices $S=[\begin{array}{cc} S_{11} & S_{12}\\ S_{12}^{T} & S_{22} \end{array}]:$

(1) S<0;
(2) $S_{11}<0,S_{22}-S_{12}^{T}S_{11}^{-1}S_{12}<0;$
(3) $S_{22}<0,S_{11}-S_{12}S_{22}^{-1}S_{12}^{T}<0$.

***Lemma 2*** [27]: Given a circular region D(o,r), the eigenvalues of a matrix $A\in R^{n\times n}$ belong to D(o,r), if there exists a symmetric matrix $P\in R^{n\times n}>0$ such that


$$[\begin{array}{cc} -P & PA-oP\\ {}* & -r^{2}P \end{array}]<0$$ (3)

where o+j0 is the center and r is the radius.

According to Assumptions 1 and 2, a robust controller based on the state estimation is designed for the nonlinear discrete interconnected system (1), so as to achieve robust stabilization of the interconnected system. In the method proposed in this paper, the observer and controller are designed separately. First, the state estimates of each subsystem are obtained through the state observer. Then, the controller is designed according to the obtained state information to make the nonlinear closed-loop control system robust.

## 3. State observer design for interconnected systems

To perform a robust estimation of the state of the interconnected system (1) and finally realize feedback control based on the estimated state, the following form of the state observer was designed:


$$\left\{ \begin{array}{c} {\hat{x}}_{i}(k+1)=A_{i}{\hat{x}}_{i}(k)+B_{i}u_{i}(k)+g\left[{\hat{x}}_{i}(k)\right]+\sum_{\begin{array}{c} j=1\\ j\neq i \end{array}}^{N}H_{ij}{\hat{x}}_{j}(k)+L_{i}[y_{i}(k)-{\hat{y}}_{i}(k)]\;\\ {\hat{y}}_{i}(k)=C_{i}{\hat{x}}_{i}(k),i=1,2,\cdots ,N.\; \end{array}\;\right.\;$$ (4)

where ${\hat{x}}_{i}(k)\in R^{n}$,${\hat{y}}_{i}(k)\in R^{p}$ are the estimated values of the state and output vectors of the i-*th* subsystem, and $L_{i}$ is the observer gain matrix of the first subsystem to be designed.

***Remark 2:*** In the design of the observer, the goal is to realize a robust estimation of the interconnected subsystems by designing the state observer. The design of the observer should consider the influence of perturbation on the state estimation, as well as the performance of the observer, to provide as effective a state estimation as possible for the design of the final controller.

With the state estimate error defined as $e_{i}(k)=x_{i}(k)-{\hat{x}}_{i}(k)$, the dynamic equation of the estimation error of each subsystem observer is


$$e_{i}(k+1)=(A_{i}-L_{i}C_{i})e_{i}(k)+\Delta g_{i}(k)+\underset{\begin{array}{c} j=1\\ j\neq i \end{array}}{\sum^{N}}H_{ij}e_{j}(k)+D_{i}d_{i}(k),i=1,2,\cdots ,N.$$ (5)

Where $\Delta g_{i}(k)=g\left[x_{i}(k)\right]-g\left[{\hat{x}}_{i}(k)\right]$.

In the design of the system observer, to provide sufficient conditions for the existence of an interconnected system state observer, the error dynamic equation (5) can be written in the following global description form:


e(k+1)=(A−LC+H)e(k)+Δg(k)+Dd(k) (6)

Where


$$e(k)=[e_{1}^{T}(k),e_{2}^{T}(k),\cdots ,e_{N}^{T}(k){]}^{T};$$

$$A=diag\left(A_{1},A_{2},\cdots ,A_{N}\right);$$

$$L=diag\left(L_{1},L_{2},\cdots ,L_{N}\right);$$

$$C=diag\left(C_{1},C_{2},\cdots ,C_{N}\right);$$

$$D=diag\left(D_{1},D_{2},\cdots ,D_{N}\right);$$

$$H=[\begin{array}{cc} \begin{array}{cc} 0 & H_{12}\\ H_{21} & 0 \end{array} & \begin{array}{cc} \cdots & H_{1N}\\ \cdots & H_{2N} \end{array}\\ \begin{array}{cc} \vdots & \vdots \\ H_{N1} & H_{N2} \end{array} & \begin{array}{cc} \ddots & \vdots \\ \cdots & 0 \end{array} \end{array}];$$

$$\Delta g(k)=[{\Delta g}_{1}^{T}(k),{\Delta g}_{2}^{T}(k),\cdots ,{\Delta g}_{N}^{T}(k){]}^{T};$$

$$d(k)=[d_{1}^{T}(k),d_{2}^{T}(k),\cdots ,d_{N}^{T}(k){]}^{T};$$

And, according to (2), we can get


$$\left\|\Delta g(k)\right\|\leq L_{g}\left\|e(k)\right\|,L_{g}=\max\left(L_{g_{1}},L_{g_{2}},\cdots ,L_{g_{N}}\right).$$

***Remark 3:*** By defining global variables and global matrices, the discrete interconnected error system equation (5) can be expressed by an augmented global system equation (6), which avoids the influence of interconnection terms in the system on the error system analysis and facilitates the stability study of the error system. Finally, sufficient conditions for the existence of an observer gain matrix of the interconnected system are provided.

To stabilize the error-dynamic system (6) in the shortest possible time and simultaneously satisfy the $H_{\infty }$ performance $\left\|e(k)\right\|\leq \gamma_{1}\left\|d(k)\right\|\;and\;\gamma_{1}>0$, the following multi-constraint design of the gain matrix L is carried out to improve the performance of the state observer:

***Theorem 1*** If there exist symmetric positive definite matrices $P=diag(P_{1},P_{2},\cdots ,P_{N})$, then matrices $X=diag(X_{1},X_{2},\cdots ,X_{N})$, disks $D(o_{1},r_{1})$, and scalars $\varepsilon_{1}>0,\gamma_{1}>0,\sigma >0$, such that the linear matrix inequalities [7,8], and (9) hold:


$$[\begin{array}{ccccc} -P+\varepsilon_{1}L_{g}^{2}I & A^{T}P-C^{T}X^{T}+H^{T}P & A^{T}PD-C^{T}X^{T}D+H^{T}PD & A^{T}P-C^{T}X^{T}+H^{T}P & I\\ {}* & P-\varepsilon_{1}I & PD & 0 & 0\\ {}* & * & D^{T}PD-\gamma_{1}I & 0 & 0\\ {}* & * & * & -P & 0\\ {}* & * & * & * & -\gamma_{1}I \end{array}]<0$$ (7)

$$[\begin{array}{cc} -P & PA-XC+PH-o_{1}P\\ {}* & -r_{1}^{2}P \end{array}]<0$$ (8)

$$[\begin{array}{cc} -(1-\sigma )P & A^{T}P-C^{T}X^{T}+H^{T}P\\ {}* & -P \end{array}]<0$$ (9)

where


X=PL;

$$I=I_{N}⊗I_{n}.$$

Then, the eigenvalues of the matrix A−LC+H lie in the disk $D(o_{1},r_{1})$ and the error system (6) satisfies the performance $\left\|e(k)\right\|\leq \gamma_{1}\left\|d(k)\right\|\;$; the observer gain matrix of each subsystem $L_{i}$is obtained as $L_{i}=P_{i}^{-1}X_{i},i=1,2,\cdots ,N.$

***Proof:*** According to Lemma 2, the eigenvalues of matrix A−LC+H lie in disk $D(o_{1},r_{1})$ and the following inequality holds:


$$[\begin{array}{cc} -P & PA-PLC+PH-o_{1}P\\ {}* & -r_{1}^{2}P \end{array}]<0$$ (10)

Let X=PL, then


$$[\begin{array}{cc} -P & PA-XC+PH-o_{1}P\\ {}* & -r_{1}^{2}P \end{array}]<0$$ (11)

De-representing the matrix in (11) in chunks yields (8), and the position of the pole assignment of the error system is restricted.

Define the following Liapunov function:


$$V_{1}(k)=\underset{i=1}{\sum^{N}}{e_{i}^{T}(k)P}_{i}e_{i}(k)=e^{T}(k)Pe(k)$$ (12)

According to (6),


$$\begin{array}{cc} \Delta V_{1}(k) & =e^{T}(k+1)Pe(k+1)-e^{T}(k)Pe(k)\\ & =[(A-LC+H)e(k)+\Delta g(k)+Dd(k){]}^{T}P[(A-LC+H)e(k)+\Delta g(k)+Dd(k)]-e^{T}(k)Pe(k)\\ & =e^{T}(k)[(A-LC+H{)}^{T}P(A-LC+H)-P]e(k)+2e^{T}(k)(A-LC+H{)}^{T}P\Delta g(k)\\ & \;+2e^{T}(k)(A-LC+H{)}^{T}PDd(k)+2\Delta g^{T}(k)PDd(k)+\Delta g^{T}(k)P\Delta g(k)+d^{T}(k)D^{T}PDd(k) \end{array}$$ (13)

According to Assumption 2, the nonlinear part satisfies the Lipschitz condition, and for arbitrary constant $\varepsilon_{1}$>  0, the following inequality exists:


$$\Delta g(k)\leq L_{g}\left\|e(k)\right\|$$

$$\varepsilon_{1}[L_{g}^{2}e^{T}(k)e(k)-\Delta g^{T}(k)\Delta g(k)]\geq 0\;$$ (14)

From (13) and (14), it is known that.


$$\begin{array}{cc} \Delta V_{1}(k) & \leq e^{T}(k)[(A-LC+H{)}^{T}P(A-LC+H)-P]e(k)+2e^{T}(k)(A-LC+H{)}^{T}P\Delta g(k)+2e^{T}(k)(A-LC+H{)}^{T}PDd(k)\\ & \;+2\Delta g^{T}(k)PDd(k)+\Delta g^{T}(k)P\Delta g(k)+d^{T}(k)D^{T}PDd(k)+\varepsilon_{1}[L_{g}^{2}e^{T}(k)e(k)-\Delta g^{T}(k)\Delta g(k)] \end{array}$$ (15)

To minimize the effect of perturbations on the state estimation such that (6) meets the performance specification $\left\|e(k)\right\|\leq \gamma_{1}\left\|d(k)\right\|\;$, the following must be satisfied:


$$J_{1}=\underset{k=0}{\sum^{\infty }}[\frac{1}{\gamma_{1}}e^{T}(k)e(k)-\gamma_{1}d^{T}(k)d(k)]<0$$

From (12) and (14), it is known that


$$\begin{array}{cc} J_{1} & \leq \sum_{k=0}^{\infty }[\Delta V_{1}(k)+\varepsilon_{1}L_{g}^{2}e^{T}(k)e(k)-\varepsilon_{1}\Delta g^{T}(k)\Delta g(k)+\frac{1}{\gamma_{1}}e^{T}(k)e(k)-\gamma_{1}d^{T}(k)d(k)]\\ & =\sum_{k=0}^{\infty }\{e^{T}(k)[(A-LC+H{)}^{T}P(A-LC+H)-P\\ & \;+\frac{1}{\gamma_{1}}I+\varepsilon_{1}L_{g}^{2}I]e(k)+2e^{T}(k)(A-LC+H{)}^{T}P\Delta g(k)+2e^{T}(k)(A-LC+H{)}^{T}PDd(k)+2\Delta g^{T}(k)PDd(k)\\ & \;+\Delta g^{T}(k)(P-\varepsilon_{1}I)\Delta g(k)+d^{T}(k)D^{T}PDd(k)-\gamma_{1}d^{T}(k)d(k)\}=\sum_{k=0}^{\infty }[\begin{array}{c} e(k)\\ \Delta g(k)\\ d(k) \end{array}{]}^{T}\Theta [\begin{array}{c} e(k)\\ \Delta g(k)\\ d(k) \end{array}]. \end{array}$$

where


$$\Theta =[\begin{array}{ccc} (A-LC+H{)}^{T}P(A-LC+H)-P+\frac{1}{\gamma_{1}}I+\varepsilon_{1}L_{g}^{2}I & (A-LC+H{)}^{T}P & (A-LC+H{)}^{T}PD\\ {}* & P-\varepsilon_{1}I & PD\\ {}* & * & D^{T}PD-\gamma_{1}I \end{array}]$$ (16)

Thus, the sufficient condition for (6) to satisfy the performance index $\left\|e(k)\right\|\leq \gamma_{1}\left\|d(k)\right\|\;$ is Θ<0, and using twice Schur’s complementary lemma, the matrix inequality Θ<0 is equivalent to the following inequality:


$$[\begin{array}{ccccc} -P+\varepsilon_{1}L_{g}^{2}I & (A-LC+H{)}^{T}P & (A-LC+H{)}^{T}PD & (A-LC+H{)}^{T}P & I\\ {}* & P-\varepsilon_{1}I & PD & 0 & 0\\ {}* & * & D^{T}PD-\gamma_{1}I & 0 & 0\\ {}* & * & * & -P & 0\\ {}* & * & * & * & -\gamma_{1}I \end{array}]<0$$ (17)

and


$$[\begin{array}{ccccc} -P+\varepsilon_{1}L_{g}^{2}I & A^{T}P-C^{T}X^{T}+H^{T}P & A^{T}PD-C^{T}X^{T}D+H^{T}PD & A^{T}P-C^{T}X^{T}+H^{T}P & I\\ {}* & P-\varepsilon_{1}I & PD & 0 & 0\\ {}* & * & D^{T}PD-\gamma_{1}I & 0 & 0\\ {}* & * & * & -P & 0\\ {}* & * & * & * & -\gamma_{1}I \end{array}]<0$$ (18)

where X=PL.

Describing the matrices in the inequality (18) using chunking matrices is equivalent to (7).

To shorten the convergence time of the state estimation error system, so that the estimate of the state observer tends to the true value as soon as possible, we reconstrain the gain matrix, which is the inequality (9) constructed, and strive to achieve a better estimation effect. Thus, when inequalities (7)–(9) are satisfied, a high-performance observer of interconnected systems can be obtained, and Theorem 1 is proved.

***Remark 4:*** In Theorem 1, inequality (7) can ensure that the error system formula (6) meets the performance criterion $\left\|e(k)\right\|\leq \gamma_{1}\left\|d(k)\right\|\;$; inequality (8) constrains the eigenvalues of the augmented matrix A−LC+H through the disk lemma $D(o_{1},r_{1})$, so that they are distributed in the disk, ensuring the stability of the error system; and inequality (9) can set the values of constants σ, thus further improving the performance of the observer.

In the next step, the controller for the nonlinear discrete interconnected system is constructed on the basis of the state observer designed in the second part to realize robust calibration of the feedback closed-loop control system.

## 4. Controller design of an interconnected system based on observed state

Based on the system state estimate obtained from the state observer (4), the following robust controller is designed:


$$u_{i}(k)=K_{i}{\hat{x}}_{i}(k)$$ (19)

According to (1) and (19), the following closed-loop control system can be obtained:


$$x_{i}(k+1)=(A_{i}+B_{i}K_{i})x_{i}(k)-B_{i}K_{i}e_{i}(k)+\underset{\begin{array}{c} j=1\\ j\neq i \end{array}}{\sum^{N}}H_{ij}x_{j}(k)+g\left[x_{i}(k)\right]+D_{i}d_{i}(k),i=1,2,\cdots ,N.$$ (20)

Similarly, the complex interconnection term in (20) can be avoided during the design of the gain matrix of the interconnected system controller by representing the feedback closed-loop interconnected system (20) as a feedback closed-loop global system of the following form:


x(k+1)=(A+BK+H)x(k)+g[x(k)]+Wη(k) (21)

where


$$A=diag\left(A_{1},A_{2},\cdots ,A_{N}\right);$$

$$H=[\begin{array}{cccc} 0 & H_{12} & \cdots & H_{1N}\\ H_{21} & 0 & \cdots & H_{2N}\\ \vdots & \vdots & \ddots & \vdots \\ H_{N1} & H_{N2} & \cdots & 0 \end{array}];$$

$$W=[\begin{array}{cc} -BK & D \end{array}];$$

and


$$B=diag\left(B_{1},B_{2},\cdots ,B_{N}\right);$$

$$K=diag\left(K_{1},K_{2},\cdots ,K_{N}\right);$$

$$D=diag\left(D_{1},D_{2},\cdots ,D_{N}\right);$$

$$\eta (k)=[\begin{array}{cc} e^{T}(k) & d^{T}(k) \end{array}{]}^{T};$$

$$e(k)=[e_{1}^{T}(k),e_{2}^{T}(k),\cdots ,e_{N}^{T}{(k)]}^{T};$$

$$d(k)=[d_{1}^{T}(k),d_{2}^{T}(k),\cdots ,d_{N}^{T}(k){]}^{T};$$

$$g[x(k)]=[g_{1}^{T}\left[x(k)\right],g_{2}^{T}\left[x(k)\right],\cdots ,g_{N}^{T}\left[x(k)\right]{]}^{T};$$

$$x(k)=[x_{1}^{T}(k),x_{2}^{T}(k),\cdots ,x_{N}^{T}(k){]}^{T}.$$

By assumption 2, we know that


$$\left\|g[x(k)]\right\|\leq L_{g}\left\|x(k)\right\|$$ (22)

where


$$L_{g}=\max\left({L_{g}}_{1},{L_{g}}_{2},\cdots ,{L_{g}}_{N}\right).$$

According to (22), the following inequality can be obtained:


$$\varepsilon_{2}[L_{g}^{2}x^{T}(k)x(k)-g^{T}[x(k)]g\left[x(k)\right]\geq 0,\;\varepsilon_{2}>0.$$ (23)

To make the closed-loop system (21) robustly tranquilized and simultaneously satisfy the performance criterion $\left\|x(k)\right\|\leq \gamma_{2}\left\|\eta (k)\right\|$, sufficient conditions for the existence of the controller are derived as follows.

***Theorem 2*** If there exist symmetric positive definite matrices $Q=diag\left(Q_{1},Q_{2},\cdots ,Q_{N}\right)$, matrix $Y=diag\left(Y_{1},Y_{2},\cdots ,Y_{N}\right)$, disk ${D(o}_{2},r_{2})$, constants $\varepsilon_{2}>0$ and $\gamma_{2}>0$ such that the following inequalities hold:


$$[\begin{array}{cccccc} -Q & QA+BY+QH & Q & -BY & QD & 0\\ {}* & -Q+\varepsilon_{2}L_{g}^{2}I & 0 & 0 & 0 & I\\ {}* & * & -\varepsilon_{2}I & 0 & 0 & 0\\ {}* & * & * & -\gamma_{2}I & 0 & 0\\ {}* & * & * & * & -\gamma_{2}I & 0\\ {}* & * & * & * & * & -\gamma_{2}I \end{array}]<0$$ (24)

$$[\begin{array}{cc} -Q & QA+BY+QH-o_{2}Q\\ {}* & -r_{2}^{2}Q \end{array}]<0$$ (25)

where


QBK=BY;

$$I=I_{N}⊗I_{n}.$$

Then, the eigenvalues of the matrix A+BK+H are located at disk ${D(o}_{2},r_{2})$ and the closed-loop control system (21) satisfies the performance criterion $\left\|x(k)\right\|\leq \gamma_{2}\left\|\eta (k)\right\|$, and the subsystem controller gain matrix $K_{i}$ is obtained as $K_{i}=B_{i}^{+}Q_{i}^{-1}B_{i}Y_{i},i=1,2,\cdots ,N$.

***Proof:*** According to Lemma 2, the eigenvalues of matrix A+BK+H lie in disk ${D(o}_{2},r_{2})$ and the following inequality holds:


$$[\begin{array}{cc} -Q & QA+QBK+QH-o_{2}Q\\ {}* & -r_{2}^{2}Q \end{array}]<0$$ (26)

Let QBK=BY, then


$$[\begin{array}{cc} -Q & QA+BY+QH-o_{2}Q\\ {}* & -r_{2}^{2}Q \end{array}]<0$$ (27)

De-representing the matrix in (27) in chunks yields (25).

Define the Lyapunov function:


$$V_{2}(k)=\underset{i=1}{\sum^{N}}{x_{i}^{T}(k)Q}_{i}x_{i}(k)=x^{T}(k)Qx(k)$$ (28)

According to equation (21), it can be obtained that


$$\begin{array}{cc} \Delta V_{2}(k) & =x^{T}(k+1)Qx(k+1)-x^{T}(k)Qx(k)\\ & =[(A+BK+H)x(k)+g[x(k)]+W\eta (k){]}^{T}Q[(A+BK+H)x(k)+g[x(k)]+W\eta (k)]-x^{T}(k)Qx(k)\\ & =x^{T}(k)[(A+BK+H{)}^{T}Q(A+BK+H)]x(k)+2x^{T}(k)(A+BK+H{)}^{T}Qg[x(k)]\\ & \;+2x^{T}(k)(A+BK++H{)}^{T}QW\eta (k)+2g^{T}[x(k)]QW\eta (k)\\ & \;+g^{T}[x(k)]Qg[x(k)]+\eta^{T}(k)W^{T}QW\eta (k)-x^{T}(k)Qx(k) \end{array}$$ (29)

For a closed-loop system (21) to satisfy performance index 11, it is necessary that


$$J_{2}=\underset{k=0}{\sum^{\infty }}[\frac{1}{\gamma_{2}}x^{T}(k)x(k)-\gamma_{2}\eta^{T}(k)\eta (k)]<0$$

According to (21) and (23), we can get


$$\begin{array}{cc} J_{2} & \leq \sum_{k=0}^{\infty }[\Delta V_{2}(k)+\varepsilon_{2}L_{g}^{2}x^{T}(k)x(k)-\varepsilon_{2}g^{T}[x(k)]g\left[x(k)\right]+\frac{1}{\gamma_{2}}x^{T}(k)x(k)-\gamma_{2}\eta^{T}(k)\eta (k)]\\ & =\sum_{k=0}^{\infty }\{x^{T}(k)[(A+BK+H{)}^{T}Q(A+BK+H)]x(k)+2x^{T}(k)(A+BK+H{)}^{T}Qg[x(k)]\\ & \;+2x^{T}(k)(A+BK+H{)}^{T}QW\eta (k)+2g^{T}\left[x(k)\right]QW\eta (k)+g^{T}\left[x(k)\right]Qg\left[x(k)\right]+\eta^{T}(k)W^{T}QW\eta (k)-x^{T}(k)Qx(k)\\ & \;+\varepsilon_{2}L_{g}^{2}x^{T}(k)x(k)-\varepsilon_{2}g^{T}\left[x(k)\right]g\left[x(k)\right]+\frac{1}{\gamma_{2}}x^{T}(k)x(k)-\gamma_{2}\eta^{T}(k)\eta (k)\}\\ & =\underset{k=0}{\sum^{\infty }}[\begin{array}{c} x(k)\\ g[x(k)]\\ \eta (k) \end{array}{]}^{T}\prod [\begin{array}{c} x(k)\\ g[x(k)]\\ \eta (k) \end{array}]. \end{array}$$

Where


$$\prod =[\begin{array}{ccc} (A+BK+H{)}^{T}Q(A+BK+H)-Q+\frac{1}{\gamma_{2}}I+\varepsilon_{2}L_{g}^{2}I & (A+BK+H{)}^{T}Q & (A+BK+H{)}^{T}QW\\ {}* & Q-\varepsilon_{2}I & QW\\ {}* & * & W^{T}QW-\gamma_{2}I \end{array}]$$

$$=[\begin{array}{c} (A+BK+H{)}^{T}Q\\ Q\\ W^{T}Q \end{array}]Q^{-1}[\begin{array}{ccc} Q(A+BK+H) & Q & QW \end{array}]+[\begin{array}{ccc} -Q+\frac{1}{\gamma_{2}}I+\varepsilon_{2}L_{g}^{2}I & 0 & 0\\ {}* & -\varepsilon_{2}I & 0\\ {}* & * & -\gamma_{2}I \end{array}].$$

Therefore, the closed-loop system (21) satisfies the performance index $\left\|x(k)\right\|\leq \gamma_{2}\left\|\eta (k)\right\|$ when ∏<0, and using Schur’s complementary lemma, the matrix inequality ∏<0 is equivalent to the following inequality:


$$[\begin{array}{cccc} -Q & Q(A+BK+H) & Q & QW\\ {}* & -Q+\frac{1}{\gamma_{2}}I+\varepsilon_{2}L_{g}^{2}I & 0 & 0\\ {}* & * & -\varepsilon_{2}I & \vdots \\ {}* & * & * & -\gamma_{2}I \end{array}]<0$$ (30)

Thus, we can obtain the following inequality:


$$[\begin{array}{ccccc} -Q & Q(A+BK+H) & Q & -QBK & QD\\ {}* & -Q+\frac{1}{\gamma_{2}}I+\varepsilon_{2}L_{g}^{2}I & 0 & 0 & 0\\ {}* & * & -\varepsilon_{2}I & 0 & 0\\ {}* & * & * & -\gamma_{2}I & 0\\ {}* & * & * & * & -\gamma_{2}I \end{array}]<0$$ (31)

Using Schur’s Lemma again, (31) can be expressed as follows:


$$[\begin{array}{cccccc} -Q & QA+BY+QH & Q & -BY & QD & 0\\ {}* & -Q+\varepsilon_{2}L_{g}^{2}I & 0 & 0 & 0 & I\\ {}* & * & -\varepsilon_{2}I & 0 & 0 & 0\\ {}* & * & * & -\gamma_{2}I & 0 & 0\\ {}* & * & * & * & -\gamma_{2}I & 0\\ {}* & * & * & * & * & -\gamma_{2}I \end{array}]<0$$ (32)

By equating the matrices in inequalities (32)–(24) and describing them by chunked matrices, the proof of Theorem 2 is complete.

***Remark 5:*** This section provides a sufficient condition for the existence of a robust controller for interconnected systems in the form of a linear matrix inequality, which allows for a greater degree of freedom in the design of the gain matrices because the system state observer and robust controller are designed separately using different Lyapunov functions.

***Algorithm1:*** Observer-based robust controller design process:

1. Construct a state observer (4) and obtain the state observation error equation (5) according to equations (1) and (4).
2. Set the $H_{\infty }$ performance index $\gamma_{1}$, disk center and radius $o_{1}$ and $r_{1}$, stability margin σ, and scalar $\varepsilon_{1}>0$, and verify the existence of the observer according to Theorem 1.
3. If feasible solutions exist for the matrix inequality (7), equation (8), and equation (9), the observer gain matrix $L_{i}$ for each subsystem is calculated.
4. The controller (19) is designed on the basis of the observer (4), and closed-loop feedback control (20) is obtained.
5. Set the $H_{\infty }$ performance index $\gamma_{2}$, disk center and radius $o_{1}$ and $r_{2}$, and scalar $\varepsilon_{2}>0$, and verify the stability of the control system according to Theorem 2. If there are feasible solutions to equations (24) and (25), calculate the gain matrix of each subsystem controller $K_{i}$ and complete the design of each subsystem controller of the interconnected system.

In the design of a feedback controller based on the observed state, an augmentation system can be built by combining a state observer and feedback control closed-loop system. The augmentation system is considered as the research target, and the gain matrix of the state observer and controller is provided to complete the controller design.

The following two tasks are completed: (a) the sufficient conditions for the existence of another observer and controller are given in the form of Theorem 3, and (b) the two controller design methods proposed in this paper are compared.

***(a) Sufficient conditions for the existence of the controller***

According to (6) and (21), the following augmentation system can be constructed:


$$\left[\begin{array}{c} e(k+1)\\ x(k+1) \end{array}]=[\begin{array}{cc} A-LC+H & 0\\ 0 & A+BK+H \end{array}][\begin{array}{c} e(k)\\ x(k) \end{array}]+[\begin{array}{c} \Delta g(k)\\ g[x(k)] \end{array}]+[\begin{array}{ccc} D & 0 & 0\\ 0 & -BK & D \end{array}][\begin{array}{c} d(k)\\ e(k)\\ d(k) \end{array}\right]$$ (33)

The augmented system formula (33) can be written as


ς(k+1)=Λς(k)+G(k)+Zξ(k) (34)

Where


$$\Lambda =[\begin{array}{cc} A-LC+H & 0\\ 0 & A+BK+H \end{array}];$$

$$Z=[\begin{array}{ccc} D & 0 & 0\\ 0 & -BK & D \end{array}];$$

$$\varsigma (k)=[\begin{array}{c} e(k)\\ x(k) \end{array}];$$

$$G(k)=[\begin{array}{c} \Delta g(k)\\ g\left[x(k)\right] \end{array}];$$

$$\xi (k)=[\begin{array}{c} d(k)\\ e(k)\\ d(k) \end{array}].$$

To minimize the effect of perturbations on the stability of the system (34), the system must satisfy the following conditions:


‖ς(k)‖≤ε‖ξ(k)‖, ε>0.  (35)

The Lyapunov function $\Delta \overset{―}{V}(k)>0$ is constructed for the system (34), and the asymptotic stability of the system (34) can be guaranteed by maintaining the following inequality:


$$\Delta \overset{―}{V}(k)+\varsigma^{T}(k)\varsigma (k)-\varepsilon^{2}\xi^{T}(k)\xi (k)<0$$ (36)

Where


$$\Delta \overset{―}{V}(k)=\overset{―}{V}(k+1)-\overset{―}{V}(k).$$

Another sufficient condition for the existence of a system observer and controller is provided in the form of a theorem.

***Theorem 3*** Given a scalar ε>0, if there exist matrices $P=diag\left(P_{1},P_{2},\cdots ,P_{N}\right)>0$, $Q=diag\left(Q_{1},Q_{2},\cdots ,Q_{N}\right)>0$and the matrices $X=diag\left(X_{1},X_{2},\cdots ,X_{N}\right)$and $Y=diag\left(Y_{1},Y_{2},\cdots ,Y_{N}\right)$ of appropriate dimensions such that the following inequality (37) holds, then an observer-based controller (19) will achieve robust stabilization of the closed-loop system (21) with disturbance level ε by inequality (36).


$$[\begin{array}{ccccccccc} \Gamma_{11} & 0 & 0 & 0 & 0 & 0 & 0 & \Gamma_{18} & 0\\ {}* & \Gamma_{22} & 0 & 0 & 0 & 0 & 0 & 0 & \Gamma_{29}\\ {}* & * & \Gamma_{33} & 0 & 0 & 0 & 0 & \Gamma_{38} & 0\\ {}* & * & * & \Gamma_{44} & 0 & 0 & 0 & 0 & \Gamma_{49}\\ {}* & * & * & * & \Gamma_{55} & 0 & 0 & \Gamma_{58} & 0\\ {}* & * & * & * & * & \Gamma_{66} & 0 & 0 & \Gamma_{69}\\ {}* & * & * & * & * & * & \Gamma_{77} & 0 & \Gamma_{79}\\ {}* & * & * & * & * & * & * & \Gamma_{88} & 0\\ {}* & * & * & * & * & * & * & * & \Gamma_{99} \end{array}]<0$$ (37)

Where


$$\Gamma_{11}=diag\left(-P_{1},-P_{2},\cdots ,-P_{N}\right)+I_{N}⊗\left(\varepsilon^{-1}{+L}_{g}^{2}\right)I_{n};$$

$$\Gamma_{18}=[\begin{array}{cccc} \mu_{11} & \mu_{12} & \cdots & \mu_{1N}\\ \mu_{21} & \mu_{22} & \cdots & \mu_{2N}\\ \vdots & \vdots & \ddots & \vdots \\ \mu_{N1} & \mu_{N2} & \cdots & \mu_{NN} \end{array}];$$

and


$$\mu_{ii}={A_{i}^{T}P}_{i}-C_{i}^{T}X_{i},i=1,2,\cdots ,N.$$

$$\mu_{ij}=H_{ji}^{T}P_{j},i\neq j\;and\;i,j=1,2,\cdots ,N.$$

$$X_{i}=P_{i}L_{i},i=1,2,\cdots ,N.$$

$$\Gamma_{22}=diag\left(-Q_{1},-Q_{2},\cdots ,-Q_{N}\right)+I_{N}⊗\left(\varepsilon^{-1}{+L}_{g}^{2}\right)I_{n};$$

$$\Gamma_{29}=[\begin{array}{cccc} \varphi_{11}^{T} & \varphi_{12}^{T} & \cdots & \varphi_{1N}^{T}\\ \varphi_{21}^{T} & \varphi_{22}^{T} & \cdots & \varphi_{2N}^{T}\\ \vdots & \vdots & \ddots & \vdots \\ \varphi_{N1}^{T} & \varphi_{N2}^{T} & \cdots & \varphi_{NN}^{T} \end{array}];$$

and


$$\varphi_{ii}=Q_{i}A_{i}+B_{i}Y_{i},i=1,2,\cdots ,N.$$

$$\varphi_{ij}=Q_{i}H_{ij},i\neq j\;and\;i,j=1,2,\cdots ,N.$$

$$Q_{i}B_{i}K_{i}=B_{i}Y_{i},i=1,2,\cdots ,N.$$

$$\Gamma_{33}=\Gamma_{44}=I_{N}⊗I_{n}.$$

$$\Gamma_{38}=diag\left(P_{1},P_{2},\cdots ,P_{N}\right).$$

$$\Gamma_{49}=diag\left(Q_{1},Q_{2},\cdots ,Q_{N}\right).$$

$$\Gamma_{55}=\Gamma_{66}=\Gamma_{77}=I_{N}⊗(-\varepsilon )I_{n}.$$

$$\Gamma_{58}=diag\left({D_{1}^{T}P}_{1},{D_{2}^{T}P}_{2},\cdots ,{D_{N}^{T}P}_{N}\right).$$

$$\Gamma_{69}=diag\left({-Y}_{1}^{T}B_{1}^{T},{-Y}_{2}^{T}B_{2}^{T},\cdots ,{-Y}_{N}^{T}B_{N}^{T}\right).$$

$$\Gamma_{79}=diag\left({D_{1}^{T}Q}_{1},{D_{2}^{T}Q}_{2},\cdots ,{D_{N}^{T}Q}_{N}\right).$$

$$\Gamma_{88}=diag\left({-P}_{1},-P_{2},\cdots ,{-P}_{N}\right).$$

$$\Gamma_{99}=diag\left({-Q}_{1},{-Q}_{2},\cdots ,{-Q}_{N}\right).$$

The gain matrix of each subsystem state observer and controller to be solved is:


$$L_{i}=P_{i}^{-1}X_{i},K_{i}=B_{i}^{+}Q_{i}^{-1}B_{i}Y_{i},\;i=1,2,\cdots ,N.$$

***Remark 6:*** The proof of Theorem 3 is similar to that of Theorem 2, and thus the proof of Theorem 3 is omitted here.

***(b) Comparison of two controller design methods in this paper***

Theorem 3 provides sufficient conditions for the existence of a state observer and a robust controller in interconnected systems by constructing an augmented system (34). Compared to the separately designed methods of the state observer and robust controller, this method has the following limitations.

1. Based on external disturbances, the design of the observer and controller $H_{\infty }$ performance indicators is not targeted, and by augmenting the system design, the $H_{\infty }$ performance indicators are conservative.
2. Considering the convergence speed of the system, the setting of the stability margins of the observer and controller is not independent, and the setting of the stability margin by augmenting the system has certain limitations.
3. For the stability problem of the system, Theorem 3 does not use the disk stability lemma to constrain the gain matrix of each subsystem of the interconnected system twice, and the condition for robust stability of the extended system (34) can be further optimized.

In other words, the observer error system and state feedback closed-loop system are augmented systems, and the centralized design of the observer and controller gain matrix has certain limitations. The observer and controller are designed separately, which is more flexible, and is conducive to improving the performance of the observer and controller.

## 5. Simulation examples and analysis

To verify the effectiveness of the observer-based robust control method proposed in this paper, a reference is made to an interconnected system composed of two subsystems [23].

Parameters for subsystem 1:


$$A_{1}=[\begin{array}{cccc} -0.4 & 0.1 & 0 & -0.2\\ 0 & 0.2 & 0 & 0.3\\ -0.8 & 0 & -0.1 & 0.2\\ 0 & 0.1 & 0.7 & 0.2 \end{array}],B_{1}=[\begin{array}{cc} 1 & 0\\ 0 & 0\\ 0 & 1\\ 0 & 0 \end{array}],D_{1}=[\begin{array}{c} 0.01\\ 0.01\\ 0.01\\ 0.01 \end{array}],d_{2}(k)=0.01sin(k),$$

$$H_{12}=[\begin{array}{cccc} 0.3 & -0.1 & 0.1 & 0.2\\ 0.1 & 0 & 0.1 & 0.1\\ 0 & 0.1 & 0 & 0.1\\ 0.2 & 0.2 & 0.1 & 0.1 \end{array}],C_{1}=[\begin{array}{cccc} 1 & 0 & 0 & 0\\ 0 & 1 & 0 & 0\\ 0 & 0 & 1 & 0 \end{array}],g_{1}\left[x_{1}(k)\right]=[\begin{array}{c} 0\\ 0.03sinx_{11}(k)\\ 0\\ 0 \end{array}].$$

Parameters for subsystem 2:


$$A_{2}=[\begin{array}{cccc} 0.1 & -0.2 & 0 & 0\\ 0 & 0.3 & 0 & 0\\ 0.2 & 0 & -0.8 & 0.2\\ 0 & 0 & 0.1 & 0.1 \end{array}],B_{2}=[\begin{array}{cc} 0 & 1\\ 1 & 0\\ 0 & 1\\ 0 & 0 \end{array}],D_{2}=[\begin{array}{c} 0.01\\ 0.01\\ 0.01\\ 0.01 \end{array}],d_{1}(k)=0.01\sin(k),$$

$$H_{21}=[\begin{array}{cccc} 0.1 & 0 & 0 & 0\\ 0 & 0.1 & 0.1 & 0.1\\ -0.1 & 0 & 0.1 & 0.1\\ 0 & 0.2 & 0 & 0 \end{array}],C_{2}=[\begin{array}{cccc} 0 & 1 & 0 & 0\\ 0 & 0 & 1 & 0\\ 0 & 0 & 0 & 1 \end{array}],g_{2}\left[x_{2}(k)\right]=[\begin{array}{c} 0.01\sin x_{21}(k)\\ 0\\ 0.02\sin x_{24}(k)\\ 0 \end{array}].$$

1. The disc region $D\left(o_{1},r_{1}\right)=(0.5,0.5)$ is selected as a constant $L_{g}=0.03,\varepsilon_{1}=25,\gamma_{1}=1.2$, and the parameters of the observer for each subsystem are determined according to Theorem 1 as follows:


$$P_{1}=[\begin{array}{cccc} 7.3825 & -0.4471 & -3.7390 & -3.9812\\ -0.4471 & 8.3569 & -0.3082 & -0.0568\\ -3.7390 & -0.3082 & 8.6813 & 4.8740\\ -3.9812 & -0.0568 & 4.8740 & 8.2143 \end{array}],P_{2}=[\begin{array}{cccc} 11.4674 & -7.5692 & 1.1261 & -3.0504\\ -7.5692 & 20.8466 & 0.3952 & 0.1193\\ 1.1261 & 0.3952 & 2.9010 & 0.0881\\ -3.0504 & 0.1193 & 0.0881 & 8.9630 \end{array}],$$

$$X_{1}=[\begin{array}{ccc} -3.1062 & 0.1362 & -0.2005\\ 1.7377 & -0.8546 & -1.2570\\ -2.7263 & 0.2362 & -1.2828\\ -0.8398 & 0.9151 & 3.2497 \end{array}],X_{2}=[\begin{array}{ccc} -4.3553 & -1.1961 & -0.7341\\ 2.1084 & -1.0938 & 1.1095\\ 0.1673 & -2.6855 & 0.3552\\ 1.0585 & 0.4280 & -1.4159 \end{array}]$$

$$L_{1}=[\begin{array}{ccc} -0.7735 & 0.0886 & 0.1024\\ 0.1449 & -0.0977 & -0.1596\\ -0.5619 & -0.0366 & -0.5378\\ -0.1427 & 0.1754 & 0.7633 \end{array}],L_{2}=[\begin{array}{ccc} -0.4618 & -0.0350 & -0.1353\\ -0.0710 & -0.0483 & 0.0018\\ 0.2478 & -0.9069 & 0.1810\\ -0.0406 & 0.0454 & -0.2058 \end{array}].$$

**Fig 1:**
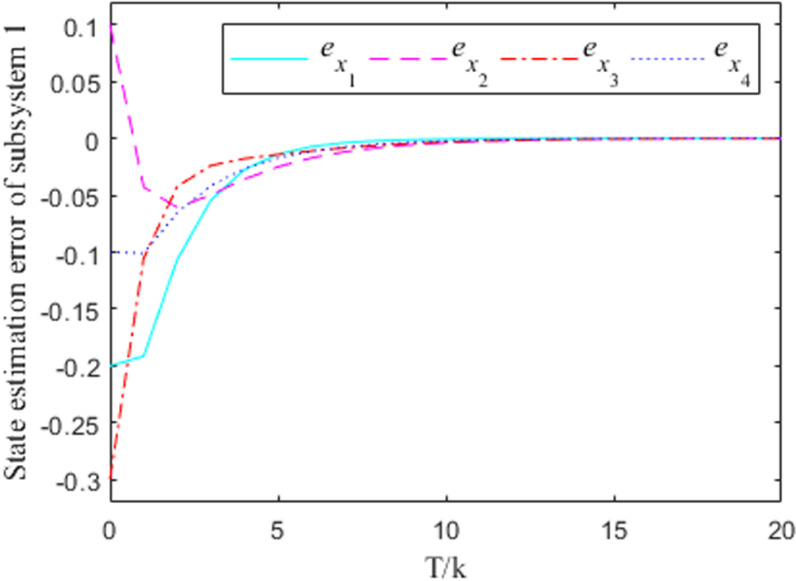
Observation error response curve for subsystem 1 when σ =  0.

**Fig 2:**
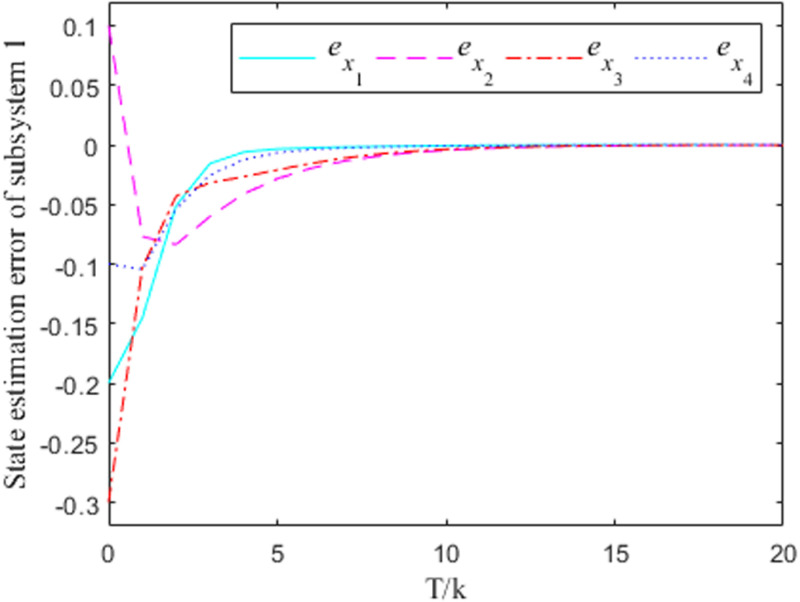
Observation error response curve for subsystem 1 when σ =  0.2.

**Fig 3:**
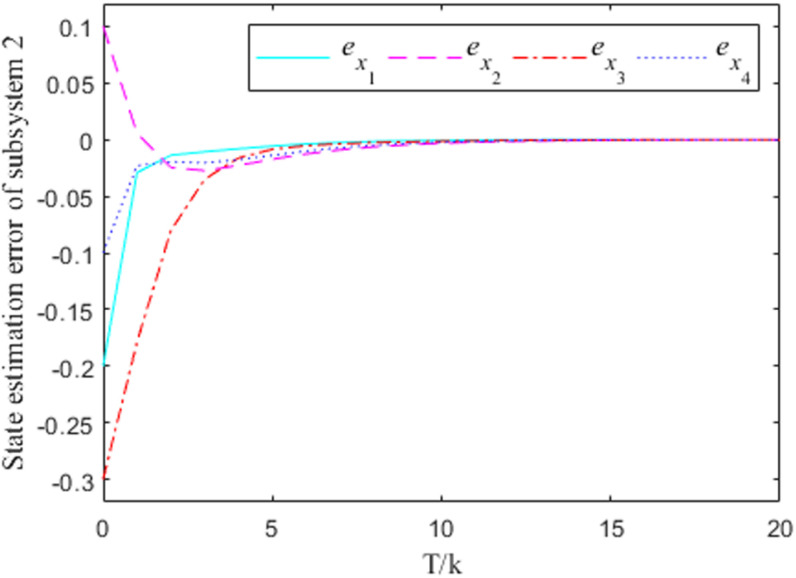
Observation error response curve for subsystem 2 when σ=0.

**Fig 4:**
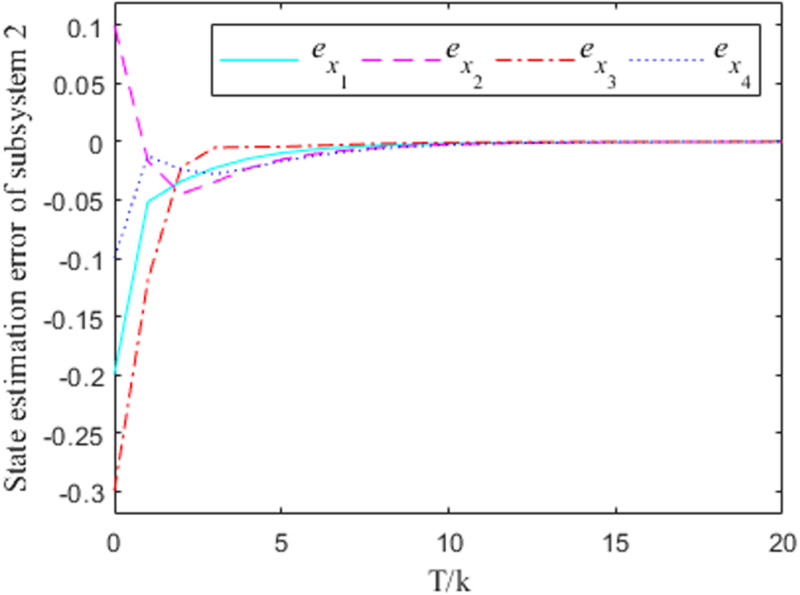
Observation error response curve for subsystem 2 when σ =  0.2.

Figs 1–4 show the response curves of the state estimation errors of the two subsystems of the interconnected system. The initial error in the state estimation of the two subsystems is $e(0)={\left(-0.2,\;0.1,-0.3,-0.1\right)}^{T}$. Because the nonlinear part of the interconnected system has little influence on the state estimation, it can be seen from the graph that the observation error of the two subsystems can essentially reach the goal of zero at the time node of k=10.

From the error curve of state estimation in the figure above, it can be seen that in subsystem 1, the estimation effect of Fig 2 is obviously better than that of Fig 1 In subsystem 2, the estimated effect of Fig 4 is better than Fig 3.

***Remark 7:*** In the controller design based on the observed state, the estimation effect of the system state is very important. In the process of designing the system state observer, the gain matrix is subjected to quadratic constraints. By shortening the time for the estimated state to approach the real state of the system, the performance and effect of the state observer are further improved, providing theoretical conditions for the final construction of the controller.

***Remark 8:*** In the process of solving the observer generalization matrix, the values of the parameters σ will have an impact on the convergence effect of the estimation error to a certain extent, and it is obvious that the case at time σ=0.2 is better than the case at time σ=0 in terms of the convergence effect. Therefore, the value of σ can be taken according to the actual situation, so as to ensure the accuracy of the state estimation. It should be noted that the σ=0.2 case is taken in the solving process of the aforementioned gain matrix.

2.The disk region $D\left(o_{2},r_{2}\right)=(0.5,0.5)$ is selected as a constant $L_{g}=0.03,\varepsilon_{2}=100,\gamma_{2}=1.5$, and the parameters of the controllers for each subsystem are determined according to Theorem 2 as follows:


$$Q_{1}=[\begin{array}{cccc} 9.4807 & 2.6541 & -9.3978 & -6.1880\\ 2.6541 & 3.5385 & -2.8228 & -1.9424\\ -9.3978 & -2.8228 & 11.9656 & 7.4890\\ -6.1880 & -1.9424 & 7.4890 & 6.7753 \end{array}],Q_{2}=[\begin{array}{cccc} 6.1435 & -3.5276 & 1.8014 & 1.9957\\ -3.5276 & 17.4614 & -0.7681 & -2.9704\\ 1.8014 & -0.7681 & 1.5407 & 0.3975\\ 1.9957 & -2.9704 & 0.3975 & 4.0971 \end{array}],$$

$$Y_{1}=[\begin{array}{cccc} 0.7930 & -0.0849 & -0.7765 & 0.9192\\ 0.6416 & 0.1507 & 1.3320 & -0.9901 \end{array}],Y_{2}=[\begin{array}{cccc} 0.7947 & -0.1629 & -0.3098 & 1.0620\\ -0.0268 & 0.2159 & 1.3510 & -0.1248 \end{array}],$$

$$K_{1}=[\begin{array}{cccc} 0.5878 & 0.0093 & 0.0692 & 0.1110\\ 0.5086 & 0.0168 & 0.1384 & 0.0266 \end{array}],K_{2}=[\begin{array}{cccc} 0.0492 & -0.0009 & 0.0393 & 0.0619\\ 0.0186 & 0.0308 & 0.2133 & 0.0102 \end{array}].$$

**Fig 5:**
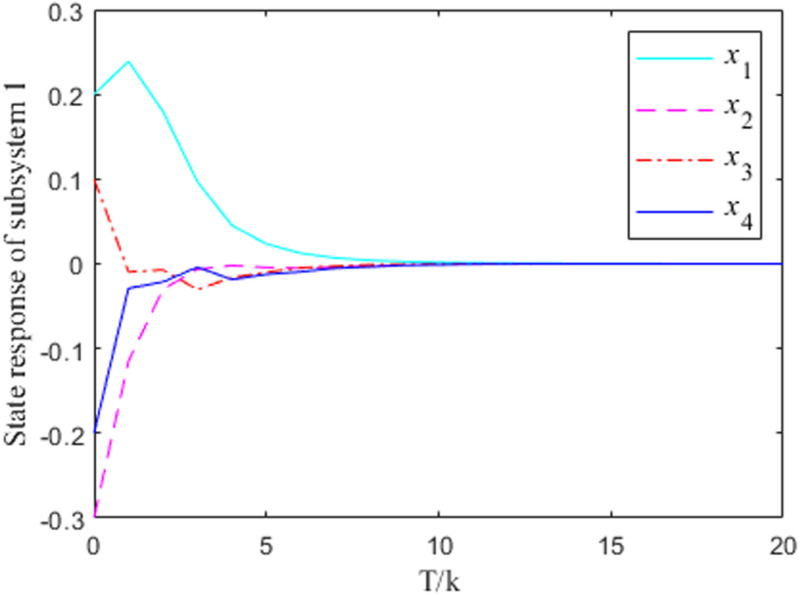
State feedback response curve of subsystem 1.

**Fig 6:**
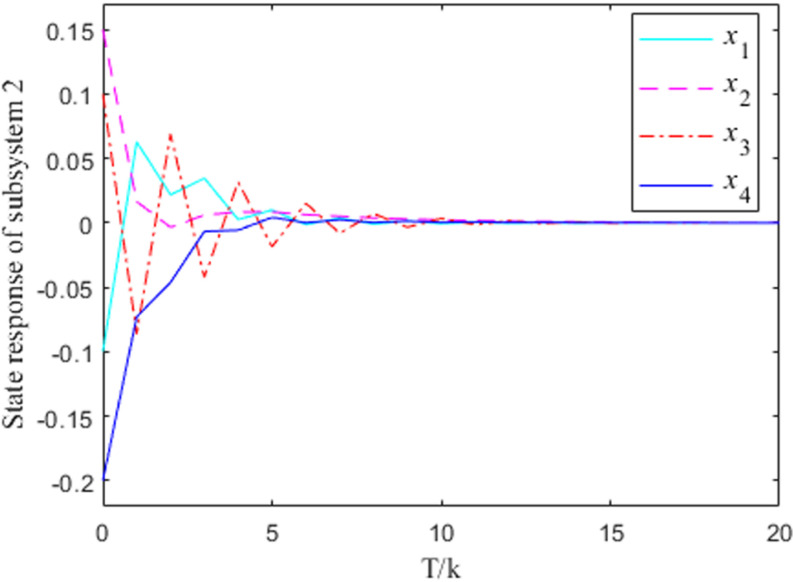
State feedback response curve of subsystem 2.

Figs 5 and 6 show the state-response curves of the two state feedback closed-loop subsystems in the interconnected system, and the initial states of the two subsystems are $x(0)={\left(-0.2,\;0.1,-0.3,-0.1\right)}^{T}$. Figs 5 and 6 show that the controller designed on the basis of the state observer can achieve a robust stabilization effect for the interconnected system, demonstrating the feasibility of the proposed control method.

## 6 . Conclusion

In this study, a robust control method based on the observed states was proposed for a class of nonlinear discrete interconnected systems with external interference. First, in the proposed method, a state observer is designed. By increasing the constraint conditions of the observer gain matrix, the convergence time of the observation error is further shortened and the performance of the state observer is improved. Second, according to the obtained state-estimation information, a robust controller is designed for the interconnected system, and sufficient conditions for the existence of the controller are provided by the disk stability lemma and Schur’s complement lemma. Finally, the robust control of the nonlinear discrete interconnected system is realized. Based on the state observer and feedback control closed-loop system, an augmentation system is constructed, and sufficient conditions for the existence of the observer and controller are given. The advantages and disadvantages of the two gain matrix solutions were analyzed. The simulation results show that the design method exhibited good convergence, and the effectiveness and feasibility of the observer-based robust control method were verified. In the study of observer-based robust control methods, the problem of robust control for nonlinear discrete interconnected systems with state delays can be considered as a future research direction.
